# Breadth of Fc-mediated effector function correlates with clinical immunity following human malaria challenge

**DOI:** 10.1016/j.immuni.2024.05.001

**Published:** 2024-05-23

**Authors:** Irene N. Nkumama, Rodney Ogwang, Dennis Odera, Fauzia Musasia, Kennedy Mwai, Lydia Nyamako, Linda Murungi, James Tuju, Kristin Fürle, Micha Rosenkranz, Rinter Kimathi, Patricia Njuguna, Mainga Hamaluba, Melissa C. Kapulu, Roland Frank, CHMI-SIKA study team, Abdirahman I. Abdi, Yonas Abebe, Philip Bejon, Peter F. Billingsley, Peter C Bull, Zaydah de Laurent, Mainga Hamaluba, Stephen L. Hoffman, Eric R. James, Melissa C. Kapulu, Silvia Kariuki, Domitila Kimani, Rinter Kimathi, Sam Kinyanjui, Cheryl Kivisi, Johnstone Makale, Kevin Marsh, Khadija Said Mohammed, Moses Mosobo, Janet Musembi, Jennifer Musyoki, Michelle Muthui, Jedidah Mwacharo, Kennedy Mwai, Francis Ndungu, Joyce M. Ngoi, Patricia Njuguna, Irene N. Nkumama, Omar Ngoto, Dennis O. Odera, Bernhards Ogutu, Fredrick Olewe, Donwilliams Omuoyo, John Ong’echa, Faith H.A. Osier, Edward Otieno, Jimmy Shangala, Betty Kim Lee Sim, Thomas L. Richie, James Tuju, Juliana Wambua, Thomas N Williams, Faith H.A. Osier

**Affiliations:** 1 Centre of Infectious Diseases, Heidelberg University Hospital, Heidelberg, Germany; 2 Centre for Geographic Medicine Research (Coast), Kenya Medical Research Institute, Wellcome Trust Research Programme, Kilifi, Kenya; 3 Epidemiology and Biostatistics Division, School of Public Health, University of the Witwatersrand, Johannesburg, South Africa; 4 Department of Biotechnology and Biochemistry, Pwani University, Kilifi, Kenya; 5 Department of Life Sciences, Imperial College London, London, UK; 6 European Vaccine Initiative, Heidelberg, Germany

## Abstract

Malaria is a life-threatening disease of global health importance, particularly in sub-Saharan Africa. The growth inhibition assay (GIA) is routinely used to evaluate, prioritize, and quantify the efficacy of malaria blood-stage vaccine candidates but does not reliably predict either naturally acquired or vaccine-induced protection. Controlled human malaria challenge studies in semi-immune volunteers provide an unparalleled opportunity to robustly identify mechanistic correlates of protection. We leveraged this platform to undertake a head-to-head comparison of seven functional antibody assays that are relevant to immunity against the erythrocytic merozoite stage of *Plasmodium falciparum*. Fc-mediated effector functions were strongly associated with protection from clinical symptoms of malaria and exponential parasite multiplication, while the gold standard GIA was not. The breadth of Fc-mediated effector function discriminated clinical immunity following the challenge. These findings present a shift in the understanding of the mechanisms that underpin immunity to malaria and have important implications for vaccine development.

## Graphical abstract

**Figure:**
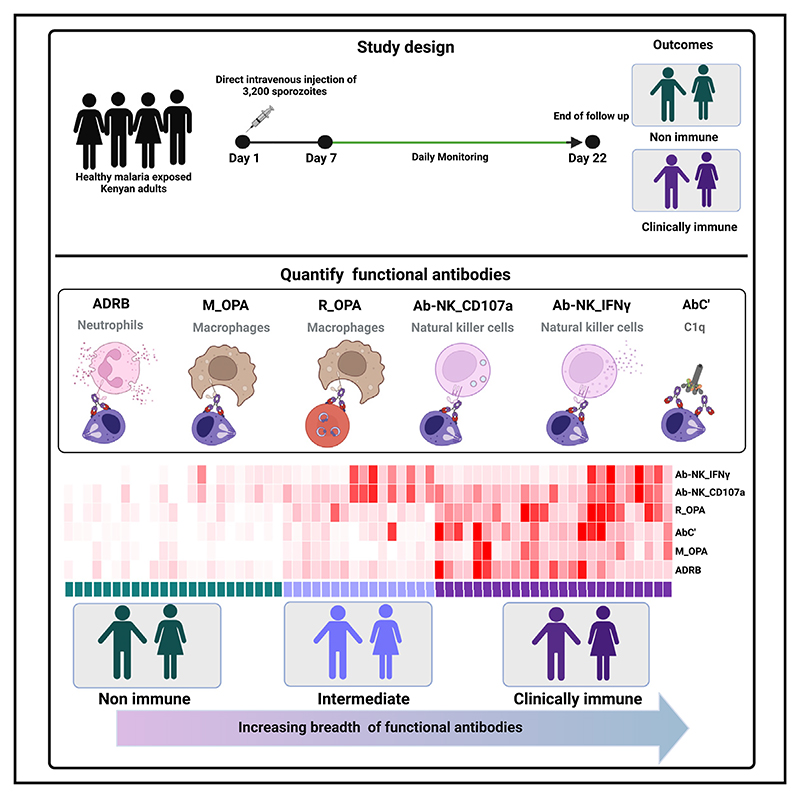

## Introduction

Malaria is caused by protozoan parasites of the phylum Apicomplexa and the genus *Plasmodium*.^1^ Multiple species of *Plasmodia* can infect humans, but *Plasmodium falciparum* (*P. falciparum*) is the deadliest and a global health priority.^2,3^ In 2022, it accounted for ~249 million clinical cases and ~608,000 deaths, mostly in sub-Saharan Africa^4^. The burden of malaria has remained high despite control efforts targeting the mosquito vector or improving parasite detection and clinical treatment.^5^ Vaccines have been challenging to develop because of the multistage life cycle of the parasite that involves different invasive forms and a complex genome encoding over 5,000 proteins, many of which are polymorphic and variably expressed.^2,6^ These difficulties are compounded by the lack of reliable immune correlates of protection.

Following decades of effort, two subunit vaccines against *P. falciparum* malaria have recently been licensed.^7–9^ Although a major achievement, challenges remain, including sub-optimal efficacy and durability of protection across diverse parasite strains. Furthermore, both vaccines target a small portion of a single protein expressed in the pre-erythrocytic sporozoite stage of the parasite life cycle.^7,9,10^ More consideration is now being given to multistage vaccines, encompassing combinations of proteins from the pre-erythrocytic, erythrocytic, and gametocyte stages. This report focuses on the erythrocytic merozoite stage, where progress in vaccine development has been largely unsuccessful, despite the fact that passive transfer studies in humans demonstrated that antibodies against the proteins expressed here were protective.^6,11^ An understanding of the mechanisms that underpin antibody-dependent protection could guide the prioritization of vaccine candidates and provide immune correlates to accelerate vaccine development.

Studies designed to understand antibody-dependent mechanisms typically focus on Fab-mediated neutralization that blocks the interaction between pathogens and host cell receptors, thereby preventing infection.^12,13^ For erythrocytic stage vaccines, this is exemplified in the growth inhibition assay (GIA), which measures the ability of antibodies to block merozoite invasion of red blood cells in culture, thus limiting parasite multiplication and growth.^14^ Although routinely used to evaluate, prioritize, and quantify the efficacy of blood-stage vaccine candidates, the GIA does not reliably predict either naturally acquired or vaccine-induced protection.^15^ Malaria vaccines inducing high levels of GIA have had limited success in clinical trials in humans.^15–19^

Antibody Fc-dependent effector functions are less well studied but appear to be increasingly important in mediating protection against infectious diseases such as HIV,^20^ Ebola,^21^ COVID-19,^22^ and malaria.^23,24^ Antibodies inducing Fc-effector function against merozoite-stage parasites are acquired with age and correlated with protection in independent cohort studies.^25–29^ However, single cellular or soluble effectors such as neutrophils,^25,30^ monocytes,^26,27^ natural killer (NK) cells,^31^ or complement^28,32^ have been analyzed in disparate studies. Neither the breadth nor the relative importance of individual Fc-effector functions have been assessed concurrently in a well-characterized experimental study of naturally acquired immunity (NAI).

We utilized the Controlled Human Malaria Infection in Semi-Immune Kenyan Adults (CHMI-SIKA) study to address these gaps, leveraging its’ distinct advantages over traditional cohort studies. First, the precise timing, strain, and dose of the inoculum are known, hence minimizing misclassification bias.^33^ Second, the close monitoring of participants in residential facilities enabled early and accurate capture of clinical outcomes, essential for safety,^34,35^ and further minimizing misclassification bias due to misdiagnoses. Third, time-dependent analyses are more robust since the timing of exposure is known, and the rapidity with which an endpoint is met is directly reflected in *in vivo* parasite growth. Consequently, the precision around the estimates of the correlates of protection is higher than that observed in cohort studies.^36^

We analyzed a panel of Fc-mediated functional assays targeting *P. falciparum* merozoites concurrently in the same study: neutrophil antibody-dependent respiratory burst (ADRB),^25^ antibody-dependent complement fixation (AbC’),^28^ antibody-mediated NK cell activation (Ab-NK),^31^ and opsonic phagocytosis of merozoites (M_OPA)^27^ and ring-stage parasites (R_OPA).^37^ We also tested the GIA^38^ and asked which assay best predicted the primary clinical outcome, defined as the need for anti-malarial treatment post-challenge.^35^ We found that Fc-mediated effector functions were correlated with protection from clinical symptoms of malaria and exponential parasite multiplication, while the gold standard GIA was not. The breadth of Fc-mediated effector function discriminated clinical immunity following challenge.

## Results

### Controlled human challenge provides clear endpoints for clinical immunity

**Figure 1:**
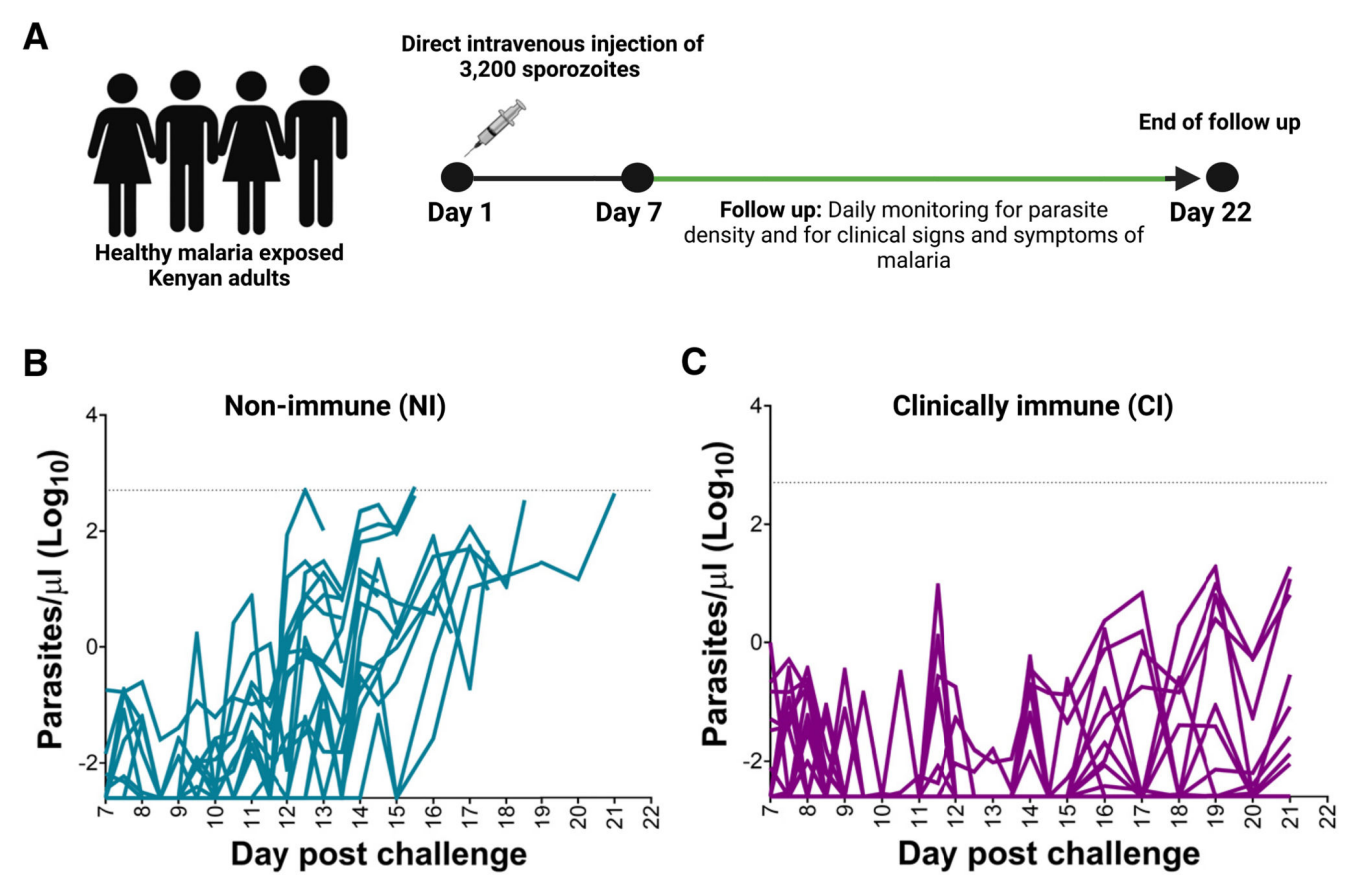
Controlled human challenge provides clear endpoints for clinical immunity (A) Study design. Kenyan adult volunteers (*N* = 64) were infected with 3,200 live *P. falciparum* sporozoites via direct venous injection. Parasitaemia was quantified by qPCR from day 7 to 21. (B) NI volunteers; parasitaemia > 500/μL of blood and/or developed fever (≥37.5°C) with any parasitaemia, *n* = 28. (C) CI volunteers; remained afebrile with parasitaemia < 500/μL, *n* = 36. The dotted line indicates the treatment threshold. See also Table S1.

One hundred and forty-two volunteers were closely monitored in residential facilities for 21 days post-challenge and eligible for analysis (Figure 1A; Table S1). However, a retrospective analysis of the blood samples collected a day before challenge (C-1) revealed the presence of the anti-malarial drugs lumefantrine or sulfadoxine (*N* = 78).^35^ Although these were below the standard minimum inhibitory concentrations,^39^ we excluded all such participants from further analysis to minimize potential confounding. Of the remaining volunteers (*N* = 64), 28 met the treatment criteria defined as parasitaemia > 500/μL or the presence of fever (>37.5°C) and any parasitaemia. These volunteers are hereafter referred to as non-immune (NI, Figure 1B). The remaining 36 volunteers did not meet the treatment criteria (parasitaemia < 500/μL and no fever) and are hereafter referred to as clinically immune (CI, Figure 1C). This reduced sample size precluded additional subgroup analyses.^35^

### Clinical immunity is associated with cytophilic anti-merozoite antibodies

We measured immunoglobulin (Ig)G, IgM, and IgG subclasses against merozoites by enzyme-linked immunosorbent assays (ELISA) in plasma samples collected 1 day before challenge (C-1). Samples were considered positive if the optical density (OD) was greater than the mean plus 3 standard deviations of malaria-naive sera.^40,41^ Antibody prevalence was defined as the proportion of all test samples that were positive. ELISAs were conducted in duplicate at a single dilution.^42,43^

**Figure 2:**
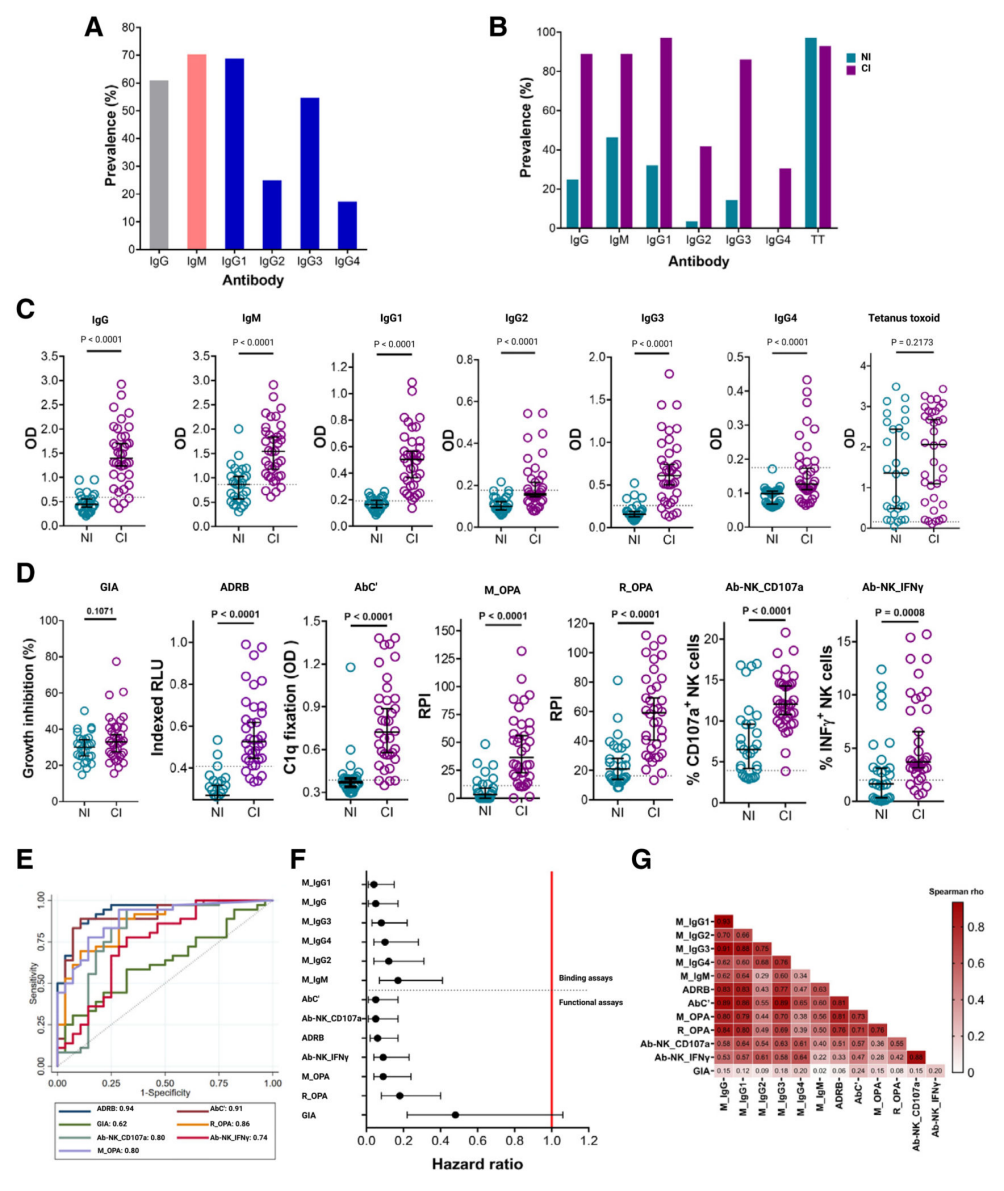
Cytophilic antibodies and Fc functions targeting merozoites are correlated with clinical immunity (A) The overall prevalence of IgG, IgM, and IgG1−4 antibodies against merozoites measured by ELISA in plasma samples collected 1 day before the challenge (C-1). (B) The prevalence of IgG, IgM, and IgG1−4 against merozoites and total IgG against tetanus toxoid extract measured by ELISA in C-1 plasma samples. (C) Quantities of IgG, IgM, and IgG1−4 antibodies against merozoites and total IgG against tetanus toxoid measured by ELISA in C-1 plasma samples. Error bars: median and 95% confidence intervals. Analysis using the Mann-Whitney U test. Seropositivity cutoff: dotted black horizontal line. (D) A comparison of plasma GIA (neutralization) and Fc-mediated effector functions in NI and CI. GIA and Fc-mediated activity measured using functional assays in C-1 plasma samples. Error bars: median and 95% confidence intervals. Analysis using the Mann-Whitney U test. Seropositivity cutoff: dotted black horizontal line. RLU, relative light units; RPI, relative phagocytosis index. (E) ROC curves for effector functions and area under the curve (AUC). (F) Forest plot showing the adjusted hazard ratios for each effector function using the Cox regression hazard model, comparing the time to treatment between volunteers with high versus low Fc-mediated function. Error bars: 95% confidence intervals. The red line indicates no protection (hazard ratio = 1.0). (G) Heatmap showing Spearman’s correlation matrix for antibody effector functions targeting merozoites and IgG, IgG subtypes (1−4), and IgM binding to merozoites (M). The color intensity indicates the strength of the correlation, while numbers indicate pairwise Spearman’s rho. See also Table S2.

Total IgG and IgM antibodies against merozoites were equally abundant in all test samples, with a prevalence of 60.9% and 70.3%, respectively (Figure 2A). Consistent with previous studies, anti-merozoite IgG subclass antibodies were predominantly cytophilic IgG1 (70.3%) and IgG3 (54.7%) (Figure 2A). Across all the isotypes measured, the antibody prevalence against merozoites was lower in the NI compared with the CI (Figure 2B). Similarly, the quantity of anti-merozoite antibodies was lower in the NI compared with the CI (*p* < 0.0001, Figure 2C). Neither the prevalence nor the quantity of total IgG to tetanus toxoid differed between groups (*p* = 0.21).

### Fc-mediated effector function is superior to neutralization in discriminating clinical immunity

We compared the ability of the GIA versus a panel of discrete Fcmediated assays to distinguish between NI and CI volunteers in C-1 samples. For the GIA, we cultured parasites of the NF54 strain used in the CHMI-SIKA study for 96 h (two cycles of parasite replication) in the presence of dialyzed and heat-inactivated plasma.^38^ For Fc-effector function, we measured ADRB, AbC’, Ab-NK, M_OPA, and R_OPA. The Ab-NK assay had two readouts: degranulation (the proportion of NK cells that were cluster of differentiation (CD) 107a^+^, AB-NK_CD107a) and interferon gamma (IFNγ) production (the proportion of NK cells that were IFNγ^+^, AB-NK_ IFNγ).^31^

There was no difference in GIA between the NI and CI, but all Fc-mediated effector functions were lower in the NI compared with CI group (*p* < 0.0001, Figure 2D). We tested this further and compared the accuracy of each effector function in predicting the clinical outcome using a receiver operating characteristic (ROC) curves analysis. Fc-mediated functions were superior to the GIA in discriminating clinical immunity, with the area under curve (AUC) between 0.74−0.94 versus 0.62, respectively (Figure 2E).

We validated our results in a Cox proportional hazards survival regression model. We dichotomized each functional assay measurement into a high versus low category using statistically derived thresholds.^40,44^ For each assay, we compared the time to treatment between volunteers with high versus low antibodies. Each Fc-mediated effector function was associated with a longer time to treatment (hazard ratios [HRs] between 0.05 to 0.19, *p* < 0.001, Figure 2F, lower panel). By contrast, the GIA was not associated with a longer time to treatment (HR 0.53, 95% CI [0.25−1.12], *p* = 0.095). The effect size for individual Fc-mediated effector functions was comparable, with an overlap between the respective confidence intervals. Gender did not modify the effect of Fc function on outcome (Table S2).

### Correlation analyses support Fc-mediated effector function over antibody binding as the key correlate of clinical immunity

Total IgG, IgM, and IgG subclass antibodies against merozoites correlated with clinical immunity (Figure 2F, upper panel). We reasoned that this was likely due to correlation between these antibodies. We found high and statistically significant pairwise correlations between total IgG, IgG1, and IgG3 (Spearman’s rho 0.93 and 0.91, respectively, *p* < 0.0001, Figure 2G). These correlations remained significant but were lower for IgG2 and IgG4 (Spearman’s rho 0.70 and 0.62, respectively, *p* < 0.0001).

Unlike the non-cytophilic IgG isotypes, cytophilic IgG1 and IgG3 isotypes are known to promote binding to Fcγ receptors.^45^ We compared the pairwise correlation coefficients between IgG1-4 and Fc-mediated functional assays. We found these correlations to be lower for the non-cytophilic isotypes (Spearman’s rho = 0.4−0.6) and higher for the cytophilic isotypes (Spearman’s rho = 0.6−0.8, Figure 2F). These data support the notion that it is Fc function and not merely antibody binding that is responsible for the clinical outcome. Furthermore, for GIA, immune effector cells are excluded, complement is deactivated through heat treatment, and plasma is dialyzed to exclude residual anti-malarial drugs using established protocols.^15^ GIA therefore only tests antibody binding and its effect on parasite growth. As shown in Figures 2G and 2F, GIA was neither associated with clinical immunity nor correlated with Fc function (Spearman’s rho < 0.25, *p* > 0.05), supporting the conclusion that Fc function rather than antibody binding per se was responsible for clinical immunity.

Next, we asked whether the antibody effector functions were correlated and found this to be true for ADRB, M_OPA, R_OPA, and AbC’ (Spearman’s rho > 0.70, *p* < 0.0001, Figure 2F). Ab-NK_CD107a and Ab-NK_IFNγ production were positively correlated (Spearman’s rho = 0.88, *p* < 0.0001). However, Ab-NK activity correlated least with other Fc-mediated effector functions (Spearman’s rho 0.28−0.57, *p* < 0.0001), suggesting the engagement of different Fcγ receptors.^45^ Overall, these data suggest that antibodies inducing Fc-mediated functions are co-acquired and may be in part polyfunctional.

Low parasite densities *in vivo* are a marker of clinical immunity, particularly in endemic areas with a high intensity of malaria transmission.^46^ We therefore tested whether Fc-mediated effector functions were more negatively correlated with parasitaemia than neutralization post-challenge. We found a weak correlation between GIA and either the mean or maximum parasite densities (Table 1). By contrast, there was a strong negative correlation between Fc-mediated effector functions with the mean and maximum parasitaemia.

### The breadth of Fc-mediated effector function discriminates clinically immune from non-immune volunteers and identifies an intermediate category

**Figure 3:**
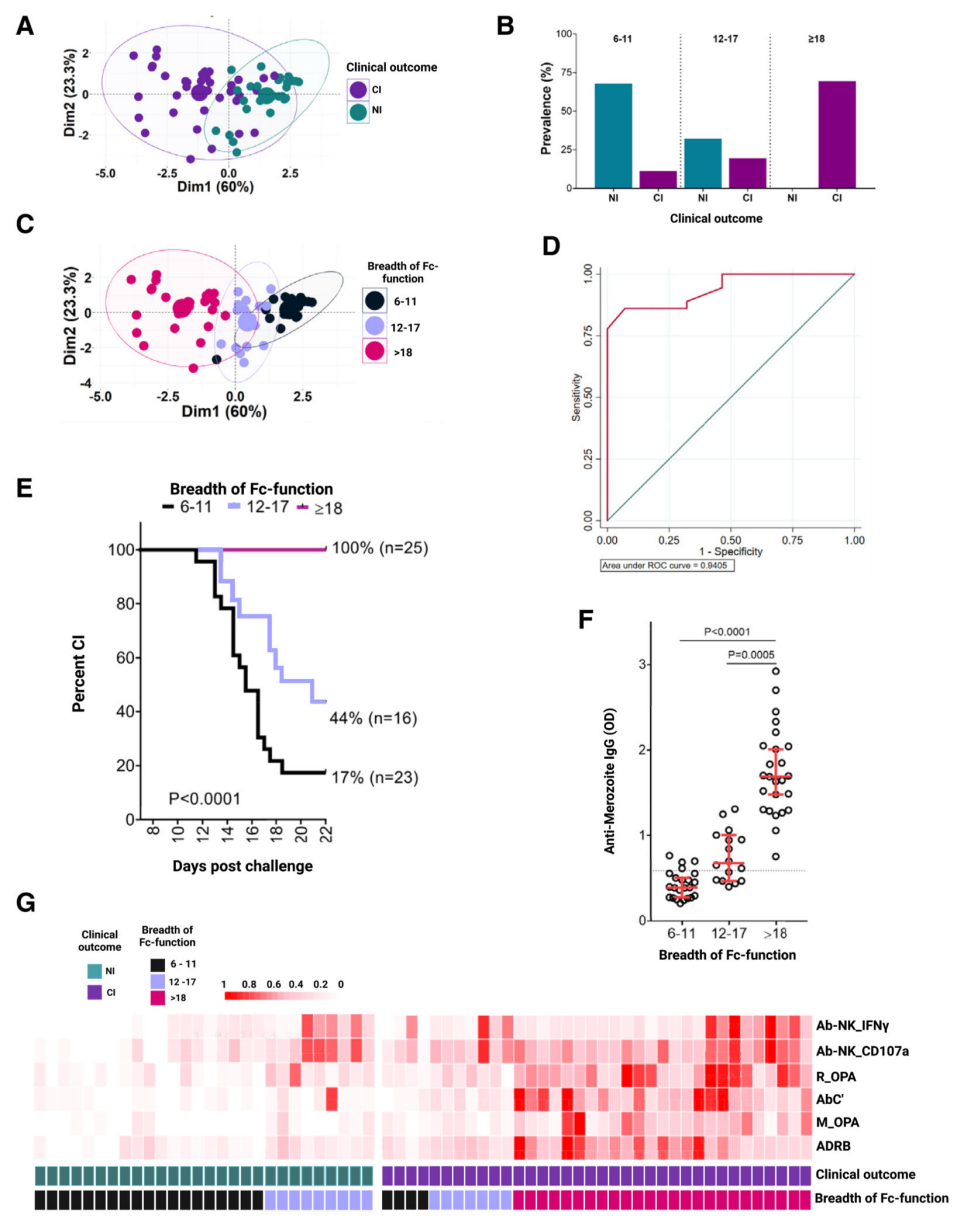
The breadth of Fc-mediated function is correlated with clinical immunity (A) PCA for the six Fc-mediated effector functions with colors representing clinical outcome. (B) The distribution of breadth scores within CI versus NI. (C) PCA for the six Fc-mediated effector functions with colors representing breadth scores. (D) ROC curve for the breadth score with AUC. (E) Survival analysis using the breadth score, showing the percentage of volunteers who were CI over time post-challenge. Each line represents the breadth of Fc function. Analysis using the log-rank (Mantel-Cox) test. (F) The quantity of anti-merozoite IgG measured in C-1 plasma samples is compared between volunteers with varying Fc breadth scores. Each dot represents an individual. Error bars: median and 95% confidence intervals. Analysis using the Kruskal-Wallis test. Seropositivity cutoff: dotted black horizontal line. (G) Heatmap of the six Fc-mediated effector functions in NI and CI volunteers. The quantity of Fc function increases from 0 to 1 and is demonstrated by darker shades of red as shown in the scale. Columns represent specific Fc-mediated functions, while rows represent individual volunteers. See also Figure S1.

We undertook a principal-component analysis (PCA) of the six Fc-mediated effector functions to visualize how well they discriminated clinical immunity. This initial analysis did not completely discriminate NI from CI volunteers (Figures 3A and S1). The breadth of the antibody response has previously been shown to be an important correlate of protection.^27,41,47–49^ We hypothesized that this failure of the PCA to discriminate CI was due to variation in the breadth of Fc function. To test this, we created a breadth score defined as the sum of responses to the six Fc-mediated functions and compared this between NIs and CIs. The full range of each immunological measure was categorized into tertiles, with corresponding scores of 1 to 4 from the lowest to the highest. The maximum score across the six Fc functions was thus 24. In keeping with our hypothesis, most of the NI volunteers (19/28, 67.8%) had a breadth score of <11, compared with CI volunteers in whom a majority (25/36, 69.4%) had a breadth score of ≥ 18 (Figure 3B). Four of thirty-six (4/36, 11.1%) CI volunteers that had breadth scores in the lowest category of between 6 and 11, while a further 7/36 (19.4%) had intermediate scores between 12 and 17. By contrast, none of the twenty-eight NI volunteers had a breadth score of ≥ 18, while 9/28 (32.1%) had intermediate scores (Figure 3B). This intermediate category was more easily visualized in a repeat PCA using the breadth score and suggests a category of individuals that at least for the NIs, are actively in the process of acquiring immunity (Figures 3C and S1). A high area under the curve of 0.94 in an ROC analysis supported the accuracy of the breadth score in predicting clinical immunity (Figure 3D).

### The breadth of Fc-mediated effector function predicts the time to clinical malaria

We tested the breadth score categories in Kaplan-Meier survival curve analysis. None of the volunteers with a breadth score of ≥ 18 developed clinical malaria during the 21 days of follow up (*n* = 25, 100% protection, Figure 3E). By marked contrast, only a proportion of volunteers with a breadth score of either 12−17 (7/16, 44%) or 6−11 (4/23, 17%) (Figure 3E) did not develop malaria. Thus, there was a significant stepwise increase in the proportion of people protected against clinical malaria, with increasing breadth from 17% to 44% to 100% (Figure 3E). Similarly, the time to clinical malaria was shorter in the group with a breadth score of 6−11, compared with 12−17 (*p* < 0.0001), and the breadth of Fc function increased with rising quantities of anti-merozoite IgG (Figure 3F), further supporting the idea that antibody binding to the merozoite was directly related to both the intensity (captured in breadth scores) and diversity (different types of Fc function, also captured in the breadth score) of Fc-effector function induced.

We explored these data further by generating a heatmap to visualize the breadth of Fc function across NI and CI individuals. It was immediately obvious that most NI individuals had large gaps in their Fc-function repertoire and consequently low breadth scores. Notably, a proportion had intermediate breadth scores, typically developed malaria toward the tail end of the study, and potentially could represent individuals actively in the process of acquiring immunity (Figures 3E and 3G). By contrast, although most of the CI volunteers had a breadth score of ≥ 18, a proportion did not, implying that additional factors contribute to immunity.

## Discussion

Passive transfer studies demonstrated the importance of antibodies in mediating protection against malaria, but the precise mechanisms that underpin immunity remain unclear. We found that individual Fc mechanisms were strongly associated with protection and superior to the well-established GIA in discriminating CI versus NI volunteers. A high breadth score of ≥ 18 that was comprised only of Fc mechanisms was completely predictive of protection from clinical malaria. Our ability to control the exposure (challenge agent) and to accurately discern who was protected versus susceptible in the CHMI-SIKA study was far higher than is typically possible in cohort studies of natural malaria infections. We consider this to be a driving factor for the compelling point estimates and narrow confidence intervals we observed in analyses of correlates of protection.

Our data compare well with those from human challenge trials following vaccination with the RTS,S malaria vaccine. Although these were conducted in malaria-naive volunteers and the analysis focused the repeat region of circumsporozoite protein (CSP) (NANP6), univariate analyses from multi-functional antibody profiling identified shared signatures of protection across independent studies, two of which were measures of Fc-mediated function: binding to FcγR3A and antibody-dependent cellular phagocytosis (ADCP).^50^ In separate studies, Fc-mediated mechanisms of immunity targeting sporozoites and involving both neutrophils and NK cells were shown to be important, particularly through FcγR3A.^51^ Other studies targeting additional stages of the malaria parasite have also found an important role for Fc-mediated mechanisms of immunity.^52^ Notably, measures of Fc function are often classified as “non-neutralizing,” even though neutralizing antibodies could also mediate Fc functions, and activity across both the Fc and Fab domains could well be synergistic.^53^ For example, invasion inhibition by anti-merozoite antibodies was increased in the presence of NK cells^31^ and complement.^28^ Similarly, neutralizing antibodies against Ebola^21^ influenza virus,^54^ HIV,^55,56^ and Hanta virus^57^ were more potent *in vivo* when they could also induce Fc-activity.

We studied a selection of Fc-mediated effector functions across three major immune cell types and C1q. A recent study found that the predominant cell types mediating phagocytosis varied depending on whether the abundance of antibody against merozoites was low or high.^58^ Notably, we used either NK cells and neutrophils from malaria-naive donors or the THP1 monocyte cell line and not the autologous cells. In Uganda, high frequencies of atypical CD56^−^ NK cells were expanded following repeated exposure to malaria and associated with clinical immunity.^59^ In addition, the expression of FcγRs on autologous leukocytes and consequently their responsiveness to IgG-mediated signaling can be modulated by inflammatory cues and differentiation status,^60^ adding a layer of complexity to data interpretation. In a similar vein, although our selection of immune cells captured the main activating and inhibitory FcγRs (Figures 1, 2A−2C, 3A, and 3B), polymorphisms in their autologous counterparts may also contribute to protection.^61^ Polymorphisms in FcγR2A (H131R) and FcγR3A (V158F) are thought to have the highest functional significance,^62^ followed by FcγR2B (I232T) and FcγR2C (Q77X). Our PCA distinguished Ab_NK (FcγR3A) from that mediated by neutrophils and monocyte responses (FcγRs Figures 1, 2A, 2B, 3B, and S1), highlighting the importance of these signaling pathways. Nevertheless, in the work presented here, our priority was to test our hypotheses by keeping the source of the immune cells constant and asking whether the variability in the quantity and quality of antibodies in the test samples was correlated with protection.

We used the NF54 parasite strain that was isolated from one of the CHMI volunteers to minimize the possibility that differences between *in vitro* GIA activity and *in vivo* parasite growth were due to the parasite strain.^6,10,63^ However, GIA was only very weakly correlated with parasitemia and was not associated with protection post-challenge. *In vitro* GIA activity was correlated with *in vivo* parasite growth in Aotus monkeys immunized with *Pf*RH5.^16^ In a recent phase 1 study, vaccination with P*f*RH5 induced antibodies with GIA activity that suggest they may be associated with clinical protection in young infants and children.^64^ Efficacy studies are eagerly awaited.

Given that our CHMI setup involved the administration of sporozoites, it remains possible that pre-erythrocytic immune mechanisms contributed to the clinical outcomes. Three distinct pieces of evidence lead us to believe that the primary mechanism of immunity is predominantly erythrocytic. First, although complete pre-erythrocytic immunity would result in sterile immunity, patent parasitaemia was observed in most volunteers.

Additional investigations in independent laboratories to ascertain the PCR-negative status of the remaining participants yielded equivocal results, suggesting these were potentially positive at the margin of detection.^35^ Thus, even in those that were apparently PCR-negative, parasites were most likely being suppressed at the blood stage. Second, alanine transferase was elevated as expected at approximately 10 days post-challenge and not different in PCR-positive versus PCR-negative volunteers, suggesting that the latter had very low parasitemia.^35^ Lastly, we considered the fact that sporozoite immunity could lower the blood-stage inoculum and thus contribute to our results. If indeed blood-stage immunity did not have a strong role, parasitaemias would have increased toward the tail end of the study.

### Limitations of the study

For safety reasons, study volunteers were treated when the parasitemia reached a predefined threshold, whether they became ill or not, and some may have been misclassified as susceptible. Notably, their breadth scores were <18, suggesting that they were not yet CI. We excluded volunteers with sickle cell trait as this is known to confer protection against malaria, but other undetermined genetic factors may be important. However, most genetic variants have a stronger impact on severe compared with mild malaria or parasitemia.^65^ Finally, antibody responses to antigens expressed on the surface of infected red blood cells^66^ or sporozoites, as well as T cell responses, Fc-receptor polymorphisms,^67–69^ and donor-specific differences in effector cell phenotypes and FcγR expression^13,70^ may have confounded our analyses and need further study.

## Star ⋆ Methods

Detailed methods are provided in the online version of this paper and include the following:

- KEY RESOURCES TABLE
- RESOURCE AVAILABILITY
  ○ Lead contact
  ○ Materials availability
  ○ Data and code availability
- EXPERIMENTAL MODEL AND STUDY PARTICIPANT DETAILS
  ○ CHMI-SIKA study
  ○ Cell lines
- METHOD DETAILS
  ○ Quantitative Polymerase Chain Reaction (qPCR)
  ○ Merozoite isolation
  ○ Anti-Merozoite ELISAs
  ○ Tetanus toxoid ELISA
  ○ Total IgG sandwich ELISAs
  ○ Total IgM sandwich ELISAs
  ○ Growth inhibition activity (GIA)
  ○ Antibody dependent complement fixation (AbC’)
  ○ Opsonic phagocytosis activity of merozoites
  ○ Phagocytosis of ring stage parasites
  ○ Antibody dependent respiratory burst (ADRB)
  ○ Antibody-mediated natural killer cells (Ab-NK) assay
- QUANTIFICATION AND STATISTICAL ANALYSIS

## Star ⋆ Methods

### Key Resources Table

**Table:** 

REAGENT or RESOURCE	SOURCE	IDENTIFIER	
Antibodies			
Donkey anti-human IgG-Fcγ fragment specific Alexafluor647 antibody	Jackson Immuno Research	Cat# 709-605-098 RRID: AB_2340577
Goat anti-human IgM − Alkaline phosphatase conjugated	Southern Biotech	Cat#2020-04 RRID: AB_2795602
Goat anti-human IgM HRP conjugate antibody	Thermo Fischer Scientific	Cat#31415 AB_228282
Mouse anti-human CD107a-PE antibody	BD Biosciences	Cat# 555801 RRID: AB_396135
Mouse anti-human CD16-APC-Cy7 antibody	BD Biosciences	Cat#561726 RRID AB_396864
Mouse anti-human CD3-PE-Cy5 antibody	BD Biosciences	Cat#555334 RRID: AB_395741
Mouse anti-human CD56-APC antibody	BD Biosciences	Cat# 341025
Mouse anti-human IFNγ-PE-Cy7 antibody	BD Biosciences,	Cat#557643 RRID:AB_396760
Peroxidase-conjugated anti-human IgG1 antibody	The Binding Site GmbH	Cat#NK006.OPT
Peroxidase-conjugated anti-human IgG2 antibody	The Binding Site GmbH	Cat#NK007.OPT
Peroxidase-conjugated anti-human IgG3 antibody	The Binding Site GmbH	Cat#LK008.OPT
Peroxidase-conjugated anti-human IgG4 antibody	The Binding Site GmbH	Cat#LK009.OPT
Purified human IgG	Sigma-Aldrich	Cat#I4506
Purified human IgM	Sigma-Aldrich	Cat#I8260
Rabbit anti-human IgG HRP antibody	Agilent	Cat#P021402-5 EAN 05700573006786
Sheep anti-human C1q, HRP conjugate antibody	Abcam	Cat#ab46191
Tetanus toxoid hyperimmune serum pool from Kenyan adults	KEMRI-Wellcome Trust Research Programme, Nduati et al.^71^	N/A
Goat anti-human IgM	Southern Biotech	Cat#2020-05 RRID: AB_2795603
Rabbit anti-human IgG	Southern Biotech	Cat#6145-01 RRID:AB_2796200
Biological samples			
Human plasma from German adults	Bloodbank Heidelberg	N/A
Human plasma from Kenyan Adults	KEMRI-Wellcome Trust Research Programme, Nduati et al.^71^	N/A
Purified human IgG from Malawian adults (MIG)	KEMRI-Wellcome Trust Research Programme, Taylor et al.^72^	N/A
Chemicals, peptides and recombinant proteins			
AlbuMAXTM I	Thermo Fischer Scientific	Cat# 11021029
Bovine Serum Albumin (BSA)	Sigma-Aldrich	Cat#A7030
Brefeldin A (BFA)	Sigma-Aldrich	Cat # B7651
Carbonate-bicarbonate capsules	Sigma-Aldrich	Cat# C3041
Casein in PBS (1% w/v)	Thermo Fischer Scientific	Cat# 37532
Dihydroethidium (DHE) dye	Abcam Limited, Cambridge	Cat#ab145360
Ethidium bromide (EtBr) dye	Thermo Fischer Scientific	Cat#15585011
SYBR green nucleic acid dye	Thermo Fischer Scientific	Cat# S7563
Trypan blue	Carl Roth, Karlsruhe	Cat# 1680.1
Circumsporozoite protein (CSP) peptide (NANP)6C	Pepscan, Germany	N/A
Complement C1q, Human	Calbiochem	Cat#204876-1MG
CountBrightTM Absolute Counting Beads	Thermo Fisher Scientific	Cat# C36950
D-Sorbitol	Sigma-Aldrich	Cat#S1876
Fetal Bovine Serum, heat inactivated	Thermo Fisher Scientific	Cat# A3840001
Paraformaldehyde (PFA)	AppliChem	Cat# 211511
Isoluminol	Santa Cruz Biotechnology	sc-485868
RPMI 1640-Medium (Gibco)	Thermo Fischer Scientific	Cat#11875093
SIGMA FAST o-Phenylenediamine dihydrochloride (OPD) tablets	Sigma-Aldrich	Cat# P9187
Tetanus toxoid TE-3	NIBSC, UK	Cat#02/232
Trans-epoxysuccinyl-L-leucylamido (4-guanidino) butane (E64)	Sigma Aldrich	Cat#E3132
Experimental models: Cells and Organisms		
Human O+ erythrocytes	Blood bank for Heidelberg University hospital	N/A
Plasmodium falciparum 3D7 strain	KEMRI-Wellcome Trust Research Programme	N/A
Plasmodium falciparum NF54 strain	KEMRI-Wellcome Trust Research Programme	N/A
THP1 cell line	ATCC, Osier et al.^27^	RRID: CVCL_0006
Software and Algorithms		
GraphPad Prism 9	GraphPad Software, Inc.	RRID: SCR_002798
STATA16	StataCorp LLC. 2019	https://www.stata.com/
R Studio v 4.2.3	R Project for Statistical Computing	https://www.R-project.org/
Flow Jo V10.9.0	BD Bioscience	RRID: SCR_008520

#### Resource Availability

##### Lead contact

Further information and requests for resources and reagents should be directed to and will be fulfilled by lead contact, Prof. Faith Osier (f.osier@imperial.ac.uk)

##### Materials availability

This study did not generate new unique reagents.

### Experimental Model And Study Participant Details

#### Chmi-Sika Study

All study volunteers were challenged under identical circumstances, in the same physical facilities, with a single identical strain, at a standardized dose, and using standardized procedures as detailed in the published study protocol.^34^ The study was open, non-blind and non-randomized. Notably, although the study subjects were all Kenyan and of similar ethnicity, they were deliberately recruited from different areas of the country to provide a diversity of previous exposures to malaria. We excluded participants with sickle cell trait as this is known to influence the outcome of malaria infections. The study was conducted in 3 phases between 2016 and 2018. One hundred and forty-two (142) participants were infected with Sanaria® 3,200 aseptic, purified, cryopreserved NF54 sporozoites by direct venous injection (DVI). Blood stage parasitemia was monitored by quantitative polymerase chain reaction (qPCR) twice a day from day 7 to day 14, and then once daily from day 15 to day 21 post challenge.^35^ The study endpoint was met when parasitaemia exceeded 500 parasites/μl of blood, or signs and symptoms of malaria developed in the presence of any parasitaemia, or at 21 days post-challenge when the study ended.^35^ Thus, our primary outcome was the need for treatment before day 21 and volunteers meeting this criterion are referred to as non-immune (NI), while those that did not are considered clinically immune (CI). We also analysed our immunological outputs against a secondary outcome of parasite density. The mean parasite density was calculated as a geometric mean of the parasite density observed between days 8.5 and day 22 post-challenge and excludes any time points after treatment. The maximum parasite density was the highest parasite density observed between the same period and similarly excluding any time points after treatment. All immunological assays presented here were conducted in plasma samples collected the day before challenge (C-1).

The CHMI-SIKA study was conducted at the Kenya Medical Research Institute (KEMRI)-Wellcome Trust Research Programme in Kilifi, Kenya, with ethical approval from the KEMRI Scientific and Ethics Review Unit (KEMRI//SERU/CGMR-C/029/3190) and by the University of Oxford Tropical Research Ethics Committee (OxTREC 2-16). All participants gave written informed consent. The study was registered on ClinicalTrials.gov (NCT02739763), conducted based on good clinical practice (GCP), and under the principles of the Declaration of Helsinki.

#### Cell lines

The human monocyte cell line, (THP-1, sourced from ATCC), was cultured in RPMI 1640 medium supplemented with 2mM L-glutamine, 10 mM HEPES, 1% pen strep (10,000 units/ml penicillin and 10,000 μg/ml streptomycin) and 10% foetal bovine serum (FBS) in a humidified incubator with 5% CO_2_ at 37°C as previously described.^27^ The cell density was monitored closely and maintained between 1 ×10^5^ and 1 ×10^6^ cells/ml. Cells were passaged when the cell density approached 1 ×10^6^ cells/ml.

### Method Details

#### Quantitative Polymerase Chain Reaction (qPCR)

The qPCR assay is published.^35^ Briefly, DNA was extracted from 500μl of whole blood and eluted in 100μl. An aliquot of 13.5μl of the DNA was used for qPCR using a TaqMan® probe (5′-FAM-AACAATTGGAGGGCAAG-NFQ-MGB-3′) that targets the multicopy 18S ribosomal RNA gene. Six serial dilutions of DNA extracted from an *in vitro* parasite culture of known parasitemia were included as standards and used to determine the parasitemia of the samples.

#### Merozoite isolation

*Plasmodium falciparum* merozoites of the NF54 or 3D7 strains were isolated as previously described.^73^ Parasites were thawed and cultured to obtain trophozoites at high parasitemia (8-12%). The trophozoites were isolated by magnetic purification and cultured in fresh complete medium to allow development to early schizont stage. A protease inhibitor, trans-epoxysuccinyl-L-leucylamido (4-guanidino) butane (E64, Sigma Aldrich), was added to allow maturation of the schizonts without rapture.^73^ Merozoites were harvested by filtration of the mature schizonts through 1.2μm filters (Pall). The merozoites were stained using either 1μg/ml ethidium bromide (EtBr, Thermo Fischer) or with 1X SYBR green dye (Thermo Fischer) for 30min and counted against CountBright™ Absolute Counting Beads (Thermo Fischer) using BD FACS Canto II flow cytometer.

#### Anti-Merozoite ELISAs

Enzyme Linked Immunosorbent Assays (ELISAs) for merozoite antigens are well-established using a standardized protocol.^27,28,40,41^ Briefly, 96-well plates (Thermo Fischer) were coated with 100μl/well of NF54 merozoites at 5x10^6^ merozoites/ml at 4°C overnight. The plates were washed and blocked with 200μl/well of blocking buffer, 1% casein (Thermo Fischer), for 2 hours at 37°C. Samples were diluted at a single dilution (1:500) with blocking buffer, added at 200μl/well and incubated for 1 hour at 37°C. Plates were washed before the addition of 100μl/well of the respective Horseradish peroxidase (HRP)-conjugated secondary antibodies: rabbit anti-human IgG (Dako), goat anti-human IgM (Southern Biotech) and rabbit anti-human IgG1, 2, 3 or 4 (The Binding Site GmbH), and incubation for 1 hour at 37°C. The plates were then washed four times and 100μl/well of substrate (o-phenylenediamine dihydrochloride (OPD), Sigma-Aldrich) was added before incubation for 20 min in the dark at room temperature (RT). The reaction was stopped with 30μl of 1M Hydrochloric acid (HCl, Sigma-Aldrich) and the absorbance measured at 492nM using a BioTek Cytation 3 cell imaging multi-mode reader. Each ELISA plate contained two sets of positive and negative controls. The positive controls were a pool of hyperimmune serum from Kenyan adults^40^ (PHIS) and a pool of purified IgG from malaria exposed adults from Malawi^72^ (MIG). The negative controls were plasma samples from malaria naïve German adults and blank wells. All assays were conducted in duplicate and the final data presented for each individual represents the mean of duplicates. Samples were re-tested afresh and in duplicate if the coefficient of variation between duplicate results exceeded 20%. The cut-off for seropositivity was defined as the mean plus 3 standard deviations of the malaria-naïve negative controls and used to estimate the antibody prevalence.

#### Tetanus toxoid ELISA

The protocol for tetanus toxoid ELISA was like the merozoite ELISA protocol above with a few variations. Briefly, plates were coated with 2μg/ml of tetanus toxoid (NIBSC), diluted in carbonate-bicarbonate buffer. Plasma samples were tested in duplicate at a 1:1000 dilution. Antibodies that bound were detected with rabbit anti-human IgG conjugated HRP (Dako). The substrate (OPD) was added and incubated for 15 min in the dark at room temperature. The reaction was stopped with 30μl of 1M Hydrochloric acid (HCL) and the absorbance measured at 492nM. Positive controls on each plate included Tetanus Immunoglublin TE-3 (National Institute for Biological Standards and Controls, UK Official Medicines Control Laboratory) and a tetanus hyperimmune serum pool from recently immunized adults from Kilifi, Kenya.^71^ Phosphate buffered saline (PBS) and blank wells were used as negative controls on each plate. The final data presented for everyone represents the mean of duplicates. Samples were re-tested afresh and in duplicate if the coefficient of variation between duplicate results exceeded 20%.

#### Total IgG sandwich ELISAs

Sandwich ELISAs to detect IgG were conducted as previously published.^71,74^ Briefly, ELISA plates (Thermo Fischer) were coated overnight at 4°C with 10μg/ml of unlabelled rabbit anti-human IgG (Southern Biotech) diluted in bicarbonate buffer. The plates were blocked with 200μl of blocking buffer (3% skimmed milk) for 1 hour at RT. Plasma samples were diluted to 1:500,000 for total IgG and incubated for 2 hours in a humidified chamber at room temperature. Antibodies that bound were detected with an HRP-conjugated rabbit anti-human IgG (Dako). The OPD substrate was added and incubated for 5-15 min in the dark at room temperature before stopping the reaction using 30μl of 1M Hydrochloric acid (HCl). The absorbance measured at 492nM. All assays were conducted in duplicate. The positive control was purified human IgG (Sigma-Aldrich), while the negative control was purified human IgM (Sigma-Aldrich). The final data presented for each individual represents the mean of duplicates. Samples were re-tested afresh and in duplicate if the coefficient of variation between duplicate results exceeded 20%.

#### Total IgM sandwich ELISAs

Sandwich ELISAs to detect IgM were similar in principle to those for IgG with minor variations.^71^ Briefly, ELISA plates (Thermo Fischer) were coated overnight at 4°C with 2.5μg/ml of unlabelled goat anti-human IgM (Southern Biotech) diluted in bicarbonate buffer. The plates were blocked with 200μl of blocking buffer (3% skimmed milk) for 1 hour at RT. Plasma samples were diluted at 1:5000. Antibodies that bound were detected using an alkaline phosphatase-conjugated goat anti-human IgM (Southern Biotech). The substrate p-Nitrophenyl Phosphate and disodium Salt (PNPP) was added and incubated for 5-15 min in the dark at room temperature. The reaction was stopped with 50 μl of 3 N NaOH and absorbance measured at 405nm. All assays were conducted in duplicate. The positive control was purified human IgM (Sigma-Aldrich), while the negative control was purified human IgG (SigmaAldrich). The final data presented for everyone represents the mean of duplicates. Samples were re-tested afresh and in duplicate if the coefficient of variation between duplicate results exceeded 20%.

#### Growth inhibition activity (GIA)

Plasma samples were dialysed using 10kDa columns (Merck Millipore GmbH) to remove any residual antimalarial drugs, and subsequently heat-treated at 56°C for 30min to inactivate complement proteins. The samples were transferred into U-bottomed 96-well plates (Thermo Fischer) at 5μl/well. Control wells contained medium only and were used as the reference to estimate inhibition as described below. Tightly synchronized trophozoite stage parasites at 0.5% and 1% haematocrit were added into each well at a volume of 45μl/well. The parasites were incubated for 96 hours to capture two cycles of parasite growth. Ten μl of fresh medium was added to each well at 48 hours. After 96 hours, the parasites were stained with 1x SYBR Green, washed and fixed in 200μl of 2% paraformaldehyde (PFA, AppliChem). The parasitemia in each well was then measured by flow cytometry using the high throughput sampler (HTS) of the BD FACS Canto II™ flow cytometer. The FACs data was analysed in FlowJo version 10 software. Percentage parasitemia was determined as the percentage of SYBR green positive erythrocytes. Percentage inhibition was calculated as: 100 (parasitemia of test sample / parasitemia of reference x 100). The positive controls were a pool of hyperimmune serum from Kenyan adults^40^ (PHIS) and a pool of purified IgG from malaria exposed adults from Malawi^72^ (MIG)) while the negative controls were plasma samples from malaria naïve German adults.

#### Antibody dependent complement fixation (AbC’)

Complement fixation was measured as previously published.^28,32^ Briefly, 96-well ELISA plates (Thermo Fischer) were coated with 100μl of freshly isolated merozoites at 5×10^6^ merozoites/ml for 2 hours at 37°C. Plates were washed and blocked with 200μl of 1% Casein in PBS for 2 hours at 37°C. Plasma samples were diluted 1:100 in 0.1% casein in PBS, added to the plates 50μl/well and incubated for 2 hours at 37°C. The plates were then washed and 40μl of human C1q (Calbiochem) was added at 10μg/ml and incubated for 30 min at RT. The bound C1q was detected using HRP-conjugated sheep polyclonal anti-C1q antibody (Abcam) diluted 1:100 for 1 hour at 37°C. The plates were washed and 100μl of OPD substrate was added and incubated for 15 min at RT before the reaction was stopped by the addition of 25μl of 2M H_2_SO_4_. Absorbance read at 492nm. Positive and negative controls were included as described for the anti-merozoite ELISAs above.

#### Opsonic phagocytosis activity of merozoites

The OPA assay is published.^27^ Briefly, THP-1 cells were cultured and diluted to 6.7×10^5^ cells/ml. 96-well U-bottom plates were precoated with 1% casein in PBS for 20 min. THP-1 cells were transferred into the plates at 150μl/well and placed in an incubator at 37°C. Separately, plasma samples were diluted 1:100 in RPMI and 10μl each was co- incubated with 100μl of ethidium bromide stained merozoites (8×10^6^merozoites/ml) for 40min at room temperature to allow opsonization to occur. For each sample, 50μl of opsonized merozoites were transferred in duplicates into the plates containing THP-1 cells and incubated at 37°C for 10 minutes to allow phagocytosis to occur.

Phagocytosis was stopped by centrifugation at 350 x g for 5 min at 4°C. Cells were washed once with 200μl of ice-cold FACS buffer (PBS + 0.5% BSA + 2mM EDTA) before resuspension in 200μl of ice-cold 2% PFA. Phagocytosis was measured using the high throughput sampler (HTS) on BD FACS Canto II™ flow cytometer. The data was analysed in FlowJo version 10 software. Phagocytosis was determined as the percentage of THP1-cells that were positive for ethidium bromide staining. A pool of hyperimmune serum from Kenyan adults (PHIS) was used as the reference sample. The relative phagocytosis index (RPI) of each sample was calculated as: phagocytosis of test sample / phagocytosis of reference sample (PHIS) X 100.

#### Phagocytosis of ring stage parasites

The OPA for ring stage parasites is published.^37^ Briefly, tightly synchronised ring stage parasites (0 10hours post-infection) at 10-15% parasitemia were stained with 5μg/ml dihydroethidium (DHE, Abcam Limited) and μM Cell Trace™ violet dye (Thermo Fischer) in PBS for 30 min at 37°C. The stained ring culture was washed twice using RPMI 1640 medium and transferred into U-bottomed 96-well plates at 0.5μl pellet/well. The ring culture was opsonised with heat-inactivated plasma diluted 1:12.5 for 30 minutes at room temperature. The opsonised cells were washed twice with RPMI 1640 and transferred into plates containing 150μl of THP1 cells (2.0 x 10^4^ cells/well) and incubated for 4 hrs at 37°C. Unphagocytosed erythrocytes were lysed using a red cell lysis buffer. The cells were then fixed with 2% PFA and phagocytosis was measured using the high throughput sampler (HTS) on the BD FACS Canto II™ flow cytometer. Phagocytosis was determined as the percentage of THP1-cells that were positive for ethidium bromide staining. A pool of hyperimmune serum from Kenyan adults (PHIS) was used as the reference sample. Previous studies demonstrated that antibody-mediated OPA was specific to the IgG fraction of malaria-exposed sera in a dose-dependent fashion.^27^ The relative phagocytosis index (RPI) of each sample was calculated as: phagocytosis of test sample / phagocytosis of reference sample (PHIS) X 100.

#### Antibody dependent respiratory burst (ADRB)

The ADRB assay is published and involved three major steps; i) parasite preparation, ii) neutrophil isolation, and iii) a chemiluminescence detection method.^75^

*Parasite preparation: Plasmodium falciparum* trophozoites of the 3D7 strain were isolated by magnetic purification and cultured in fresh complete medium to allow development to the early schizont stage. A protease inhibitor E64 (Sigma Aldrich) was added to allow the maturation of the trophozoites for 8-12 hours whilst preventing schizont rupture.^73^ The schizonts were then quantified and stored at -80°C until use. At the time of the assay, schizonts were thawed, diluted to 10 x 10^5^ schizonts/ml in PBS, and coated into white opaque 96-well plates (Greiner) at 100μl/well and incubated overnight at room temperature. The plates were then washed and blocked with 200μl/well casein in PBS for 1 hour at room temperature. The plates were washed before the addition of 50μl of diluted plasma (1:50 in 1X PBS) to each well, followed by a 1-hour incubation at 37°C.

##### Neutrophil isolation

Polymorphonuclear leucocytes (PMN) were prepared from freshly collected whole blood for each assay using a standard procedure. Forty mls of whole blood from healthy donors was mixed at a ratio of 1:1 with Hank’s Balanced Salt Solution (HBSS, Thermo Fischer). The diluted blood was carefully layered on 7.5ml aliquots of Ficoll (GE healthcare). This was then centrifuged at 600 x g for 15 min. The supernatant was carefully removed without disturbing the red cell pellet. The pellet was resuspended in 5ml of HBSS then mixed with 3% dextran at a ratio of 1:2 and incubated at room temperature in the dark for 30min. The supernatant was then carefully collected and centrifuged at 500 x g for 7min at 4°C. The supernatant was discarded, and red cell contaminants were lysed. The cells were then centrifuged at 500 x g for 7min at 4°C and the pellet resuspended in 1ml of ice cold PMN buffer (HBSS with 0.1% bovine serum albumin (BSA, Sigma-Aldrich), 1% D -glucose). The PMN count was determined using a haemocytometer and the concentration adjusted using the PMN buffer to 1.0 X 10^7^ / ml. The cells were kept on ice.

##### Chemiluminescence detection

The schizont-coated plates were washed before the addition 50μl of PMNs (1 x 10^7^ PMNs/ml) and 50μl of 0.04mg/ml isoluminol (Santa Cruz Biotechnology) per well. The plates were immediately loaded onto a plate reader and ADRB activity quantified via chemiluminescence at 450nM every two minutes for 1.5 hours, captured as the maximal relative light units (RLU). The positive controls were PHIS and MIG, while the negative controls were malaria-naïve sera from German donors as described under merozoite ELISAs above. To account for the variability of ADRB activity from PMNs from different individuals, each sample tested in duplicate using PMNs from two independent donors.^47,76^ To further minimize variability between donors, the RLU values were indexed based on the PHIS positive controls in each plate.^47,76^ Thus, the mean RLU for the PHIS control was used as the reference sample with an indexed RLU of 1.0. The indexed RLU value for each test sample was calculated as: RLU of sample / RLU of PHIS. The mean indexed RLU values using PMNs from two donors was then calculated.

#### Antibody-mediated natural killer cells (Ab-NK) assay

The antibody-mediated natural killer cells (Ab-NK) assay is published.^31^

##### Opsonization of merozoites

Briefly, 96 well culture-plates were coated with 100μl of merozoites in PBS overnight at 4°C. Plates were then washed three times with 200μl of sterile PBS and blocked with casein in PBS for 2 hours at 37°C. Plates were washed three times with sterile PBS after blocking and before the addition of 50μl/well of plasma samples diluted 1:20 in blocking buffer. Incubation proceeded for 4 hours at 37°C and plates were subsequently washed five times with sterile PBS to remove unbound plasma components.

##### Natural Killer Cell Preparation

Peripheral blood mononuclear cells (PBMCs) were harvested by density gradient centrifugation from fresh blood collected from healthy donors, resuspended in 2 ml of culture medium and the cell density quantified using a hemocytometer. NK cells were enriched from the PBMCs using a NK negative isolation kit (Miltenyi) and magnetic cell sorting using LS columns (Miltenyi) as per the manufacturer’s instructions, and stored at 1X10^5^ cells per ml.

##### Co-incubation of NK cells and opsonized merozoites

Freshly isolated NK cells (20,000 NK cells/well), 2μl/well of brefeldin A (BFA, Sigma-Aldrich), 2μl/well of monensin (Sigma) and 2.5μ/well of PE conjugated anti-human CD107a antibody (BD Biosciences) were added to the opsonised merozoites and incubated for 18 hours at 4°C. The stimulated NK cells were then transferred into V bottom 96 well plates, centrifuged at 800 x g for 5 min at 4°C and washed with FACS buffer (0.1% Sodium Azide + 1% BSA + 1 x PBS).

##### Cell staining

The viability of NK cells was assessed using eFluor 520 (ThermoFischer Scientific). NK cell surface receptors were stained using a cocktail of anti-human mAbs comprising anti- CD56 allophycocyanin (APC), anti-CD3 phycoerythrin (PE)−Cy5, and anti-CD16 APC-Cy7 (BD Biosciences) for 30 min at 4°C. The plates were then washed twice with FACS buffer, fixed with Cell Fix (BD Biosciences) for 10 mins, and permeabilized using Perm/Wash (BD Biosciences) for 10 min at 4°C. The permeabilized cells were then stained intracellularly with anti-human IFNɣ-PE-Cy7 antibody (BD biosciences) for one hour in the dark. Finally, the cells were washed with Perm/Wash, resuspended in FACS buffer, and stored at 4°C prior to data acquisition.

##### Flow cytometry

Data was acquired using a BD Biosciences FACS Calibur II high throughput instrument in a 96-well format using FACSDiva. The flow data gating strategy and analysis are published.^31,76^The assay positive controls were PHIS and MIG, while the negative controls were malaria-naïve sera from German donors as described under merozoite ELISAs above. The proportion of NK cells expressing IFNɣ and or CD107a (degranulating NK cells) were determined using FlowJo version 10.

### Quantification And Statistical Analysis

Statistical tests are indicated in the corresponding figure legends. A significance threshold of p < 0.05 was used for all tests. Continuous variables were compared using the nonparametric Mann-Whitney U test and the Kruskal-Wallis H test with Dunn’s multiple comparisons test (GraphPad Prism) as appropriate. The accuracy of antibody function in predicting clinical outcome was analysed using the area under the curve (AUC) in receiver operating curves (ROC) analyses. The relationships between variables were examined using Spearman’s rank correlations. Time to event analyses were conducted using the Cox proportional hazards regression model in STATA16. Data were visualized using principal component analysis (PCA) tools in Rv4.2.3.

## Supplementary Material

Supplementary Information

## Figures and Tables

**Table 1:** Correlations between parasitaemia and Fc-mediated effector functions

Secondary outcome variable	Mean parasitaemia				Maximum parasitaemia		
	Rho	*p*	95% confidence interval		Rho	*p*	95% confidence interval
GIA	−0.19	0.128	−0.42, 0.06		−0.27	0.031	−0.49, −0.42
ADRB	−0.73	<0.0001	−0.82, −0.58		−0.68	<0.0001	−0.79, −0.50
AbC’	−0.62	<0.0001	−0.75, −0.43		−0.59	<0.0001	−0.73, −0.40
M_OPA	−0.63	<0.0001	−0.76, −0.44		−0.61	<0.0001	−0.74, −0.42
R_OPA	−0.52	<0.0001	−0.68, −0.31		−0.50	<0.0001	−0.67, −0.28
Ab-NK_CD107a	−0.41	0.0008	−0.59, −0.17		−0.28	0.023	−0.50, −0.03
Ab-NK_IFNγ	−0.30	0.016	−0.51, −0.05		−0.16	0.199	−0.39, 0.09

## Data Availability

All data reported in this paper will be shared by the lead contact upon request. This paper does not report original code. Any additional information required to reanalyze the data reported in this paper is available from the lead contact upon request.

## Highlights

- Used a human malaria infection study to investigate functional correlates of immunity
- IgG Fc-dependent mechanisms are correlated with acquired immunity, but GIA is not
- The breadth of IgG Fc-effector function is associated with protective immunity
