# Supplementary material for: Breadth of Fc-mediated effector function correlates with clinical immunity following human malaria challenge
Source: Immunity. Author manuscript; Available in PMC 2024 Sep 28. (PMC7616646; doi:10.1016/j.immuni.2024.05.001)
Supplement: Supplementary Information [file EMS198791-supplement-Supplementary_Information.pdf]

Immunity, Volume *57*

Supplemental information

Breadth of Fc-mediated effector function correlates with clinical immunity following human malaria challenge

Irene N. Nkumama, Rodney Ogwang, Dennis Odera, Fauzia Musasia, Kennedy Mwai, Lydia Nyamako, Linda Murungi, James Tuju, Kristin Fürle, Micha Rosenkranz, Rinter Kimathi, Patricia Njuguna, Mainga Hamaluba, Melissa C. Kapulu, Roland Frank, CHMI-SIKA study team, and Faith H.A. Osier

Supplementary Information

Table S1: Baseline characteristics of the CHMI-SIKA volunteers, relates to Figure 1A

	Cohort			Total
**Year of recruitment**	**2016**	**2017**	**2018**	
**Sample size**	36	53	53	142
**Age: median (range)**	29 (18-44)	25 (20-44)	27 (18-45)	27 (18-45)
**Sec: Percentage male**	75%	73.6%	60.4%	69%
**No detectable lumefantrine**	15 (41.6%)	28 (52.8%)	31 (58.5%)	74 (52.1%)
**No detectable levels of any of the tested drugs**[a]	13 (36.1%)	23 (43.4%)	28 (52.8%)	64 (45.1%)

[a] The following antimalarial drugs were measured in plasma samples collected on day 7 post challenge: Lumefantrine, Pyrimethamine, Sulfadoxine, Chloroquine, Artesunate and Artemether. Of the 142 volunteers originally included in the study, 78 had detectable Lumefantrine or Sulfadoxine or both at levels below the minimum inhibitory concentrations (MIC, Lumefantrine 200ng/ml, Sulfadoxine 100ng/ml).

Table S2: Gender has no effect on antibody effector function, relates to figure 2F

Effector Function	Hazard Ratio Confidence Interval	P value	Gender-adjusted Hazard Ratio Confidence Interval	P value
**AbC'**	0.05 (0.01 – 0.16)	< 0.001	0.05 (0.01 – 0.17)	< 0.001
**ADRB**	0.09 (0.04 – 0.23)	< 0.001	0.10 (0.04 – 0.23)	< 0.001
**Ab-NK_CD107a**	0.09 (0.04 – 0.22)	< 0.001	0.10 (0.04 – 0.23)	< 0.001
**Ab-NK_IFNy**	0.11 (0.04 – 0.25)	<0.001	0.08 (0.03 – 0.20)	<0.001
**M_OPA**	0.12 (0.05 – 0.28)	<0.001	0.11 (0.04 – 0.27)	< 0.001
**R_OPA**	0.18 (0.08 – 0.39)	<0.001	0.17 (0.08 – 0.39)	<0.001
**GIA**	0.52 (0.24 – 1.11)	0.095	0.56 (0.25 – 1.24)	0.157

Gender did not modify the functional antibody responses. Analysis conducted using Cox proportional hazards regression models.

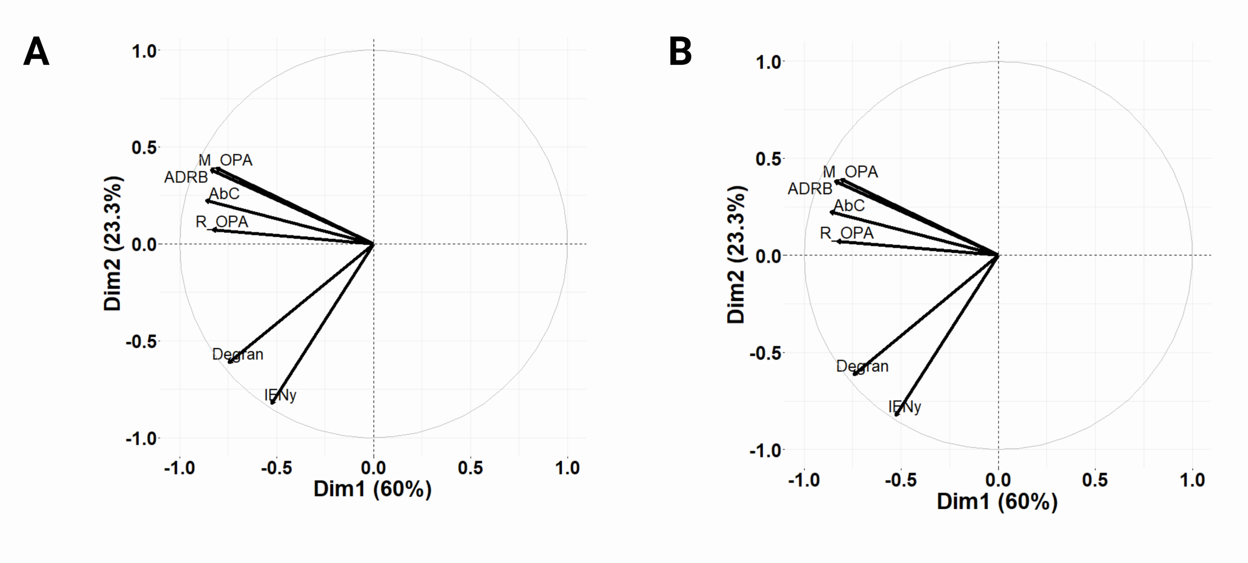

Figure S1. **The breadth of Fc-mediated function is associated with clinical immunity. Relates to Figures 3A and 3C.** Loading plot which mirrors the PCA dot plot in figure 3A (left) and 3C (right) indicating the variables that affect the distribution of the individuals in the dot plot. ADRB; antibody dependent respiratory burst by neutrophils, AbC'; complement fixation, M_OPA; opsonic phagocytosis of merozoites by monocytes, R_OPA; phagocytosis of ring stage parasites, CD107a: Fc-mediated NK cell granulation (CD107a), IFNγ: Fc-mediated NK cell IFNγ production**.**
